# PERSON-ENVIRONMENT FIT UNDERLYING PERSONALITY DEVELOPMENT IN MIDDLE AND LATE ADULTHOOD

**DOI:** 10.1093/geroni/igae098.0214

**Published:** 2024-12-31

**Authors:** Christopher Beam, Emily Schoenhofen Sharp

**Affiliations:** University of Southern California, Los Angeles, California, United States; Yale Medicine, New Haven, Connecticut, United States

## Abstract

The corresponsive principle and the niche-picking principle have been used to explain personality development across adulthood. The corresponsive principle posits that reciprocal effects between people and their environments intensify personality traits because environments are selected based on these traits while the niche-picking principle posits that these reciprocal effects simply maintain existing trait levels. Using a longitudinal sample of twins from the Swedish Adoption/Twin Study of Aging (SATSA), we tested whether the corresponsive principle or the niche-picking principle better explained longitudinal within-family differences in neuroticism, extraversion, and openness to experience across two stages of adulthood development: middle adulthood (36--56 years) and older adulthood (56--91 years). Using exploratory data analysis and multilevel structural equation modeling, we show that niche-picking rather coresponsivity tends to provide a better overall description of the development of neuroticism, extraversion, and openness in middle and older adulthood. Within families, twins generally preserve their rank-order stability but do not diverge over time. Instead, there is a generally tendency for twins to maintain within-family differences over time or even become more alike, regardless of whether twins are identical or fraternal. Nevertheless, the role that niche-picking might play in the personality development across adulthood generally is low, as the majority of the total variance of neuroticism, extraversion, and openness is attributed to stable genetic and environmental variance, particularly in older adulthood. Taken together, these findings suggest that environmental variance - whether independent of or correlated with genotype - takes on an increasingly stronger role in personality development over the lifespan.

